# Metachronal waves in the flagellar beating of *Volvox* and their hydrodynamic origin

**DOI:** 10.1098/rsif.2014.1358

**Published:** 2015-07-06

**Authors:** Douglas R. Brumley, Marco Polin, Timothy J. Pedley, Raymond E. Goldstein

**Affiliations:** 1 Department of Applied Mathematics and Theoretical Physics, Centre for Mathematical Sciences, University of Cambridge, Wilberforce Road, Cambridge CB3 0WA, UK; 2 Department of Civil and Environmental Engineering, Massachusetts Institute of Technology, 77 Massachusetts Avenue, Cambridge, MA 02139, USA; 3 Physics Department, University of Warwick, Gibbet Hill Road, Coventry CV4 7AL, UK

**Keywords:** eukaryotic flagella, metachronal waves, microhydrodynamics, synchronization

## Abstract

Groups of eukaryotic cilia and flagella are capable of coordinating their beating over large scales, routinely exhibiting collective dynamics in the form of metachronal waves. The origin of this behaviour—possibly influenced by both mechanical interactions and direct biological regulation—is poorly understood, in large part due to a lack of quantitative experimental studies. Here we characterize in detail flagellar coordination on the surface of the multicellular alga *Volvox carteri*, an emerging model organism for flagellar dynamics. Our studies reveal for the first time that the average metachronal coordination observed is punctuated by periodic phase defects during which synchrony is partial and limited to specific groups of cells. A minimal model of hydrodynamically coupled oscillators can reproduce semi-quantitatively the characteristics of the average metachronal dynamics, and the emergence of defects. We systematically study the model's behaviour by assessing the effect of changing intrinsic rotor characteristics, including oscillator stiffness and the nature of their internal driving force, as well as their geometric properties and spatial arrangement. Our results suggest that metachronal coordination follows from deformations in the oscillators' limit cycles induced by hydrodynamic stresses, and that defects result from sufficiently steep local biases in the oscillators' intrinsic frequencies. Additionally, we find that random variations in the intrinsic rotor frequencies increase the robustness of the average properties of the emergent metachronal waves.

## 1. Introduction

The eukaryotic flagellum is one of the most highly conserved structures in biology, providing locomotion and fluid transport through the execution of a periodic motion. From mucociliary clearance in the human respiratory tract [1] to cerebrospinal flows [2], and the establishment of left–right asymmetry in mammalian embryos [3], the flows generated by this periodic beating are tightly coupled to microscale transport in fundamental biological processes. Physically separated pairs of flagella can synchronize their beating purely through hydrodynamic interactions [4], but cilia and flagella usually fulfil their tasks through the concerted action of large groups, displaying a remarkably universal tendency to develop synchronous beating patterns known as metachronal waves (MWs), large-scale modulations of the beating phase [5]. Readily observed on the ciliated surface of protists such as *Opalina* and *Paramecium* [6,7] and even in synthetic bundles of active microtubules [8], MWs are still a mystery: their emergence, their properties and their biological role are poorly understood, mostly due to the difficulty of studying them *in vivo*.

Early qualitative studies of MWs [9–11] suggested a mechanical origin for synchronization, and motivated theoretical studies of hydrodynamically coupled filaments driven by different internal engines [12–16]. While these models show a general tendency towards metachronism, their complexity prevents a simple understanding of the underlying mechanism. Minimal models of hydrodynamically coupled self-sustained oscillators, instead, have the potential to offer insights into the emergence of MWs, since in many cases they admit analytical solutions which provide a direct link between model parameters and details of synchrony. Experiments with rotating paddles [17], light-driven microrotors [18], and colloids in optical tweezers [19–21], as well as simulations of rotating helices [22], or spheres driven along fixed [23–27] or flexible [28,29] trajectories, have shown that under certain conditions hydrodynamic interactions alone can induce phase-locking of two simple oscillators. This locking is mediated either through wall-modified flows [25], a variable driving force [27,30], or mechanical elasticity intrinsic in the system [28,29]. Of these, the last two provide the fastest, and possibly equivalently strong [31], drive towards synchronization. Meanwhile, experiments on the biflagellate alga *Chlamydomonas reinhardtii* [32–34], and on isolated pairs of somatic cells from the multicellular alga *Volvox carteri* [4], lend strong support to the idea that synchronization among two flagella happens indeed through the elastic response of flagella to shear stress. Even though two flagella can synchronize their motions through direct hydrodynamic interactions [4], this pair synchronization does not necessarily guarantee group synchronization, nor the emergence of MWs [28]. Owing to its large size and ease of visualization, the colonial alga *Volvox carteri* is an ideal model organism for the study of flagella-driven flows [35]. *Volvox* comprises thousands of biflagellate somatic cells, embedded within a spherical extracellular matrix shell, beating their flagella towards the colony's posterior with only a small deviation (approx. 15°) from meridian lines [36]. Here we show that these cells display a complex dynamical behaviour, with colony-wide MWs propagating towards the posterior interrupted by characteristically shaped recurrent phase defects, not previously observed in experiments. For realistic parameters, we show that a minimal model of hydrodynamically coupled identical rotors predicts MWs of wavelength similar to those observed in experiments. These MWs emerge in a manner essentially independent of boundary conditions, and are robust against realistic perturbations in the rotors' properties. Including an intrinsic frequency bias derived from experiments, the model develops periodic defects similar to those we report for *Volvox*. These findings suggest that the collective dynamics of *Volvox* flagella result from a competition between a drive towards synchronization, based on the oscillators' elastic compliance, and additional strains imposed by a large-scale bias in flagellar properties.

## 2. Metachronal waves in *Volvox carteri*

*Volvox carteri* f. *nagariensis* (strain EVE) was grown axenically in standard *Volvox* medium [37–39] bubbled with sterile air. The cultures were hosted in a growth chamber (Binder, Germany) set to a cycle of 16 h light (100 µEm^−2^ s^−1^; Fluora; OSRAM) at 28°C and 8 h dark at 26°C. Individual *Volvox* colonies within a 25 × 25 × 5 mm glass observation chamber filled with fresh medium were captured, oriented and held in place using two glass micropipettes with 100 µm diameter tips housed in pipette holders (World Precision Instruments, USA) connected to two manual microinjectors (Sutter Instruments Co., USA). Motorized micromanipulators (Patchstar, Scientifica, USA) and custom-made rotation stages allowed the pipettes to be moved in three dimensions and rotated freely along their centrelines. We aligned the axis of each colony along the focal plane of the 40× Plan Fluor lens (NA 0.6) of a Nikon TE2000-U inverted microscope, and recorded 30 s long movies of the surrounding flow with a high-speed camera (Fastcam SA3; Photron, USA) at 500 fps under brightfield illumination. A long-pass interference filter with a 10 nm transition ramp centred at 620 nm (Knight Optical, UK) prevented phototactic responses of the *Volvox* colonies.

**Figure 1.**
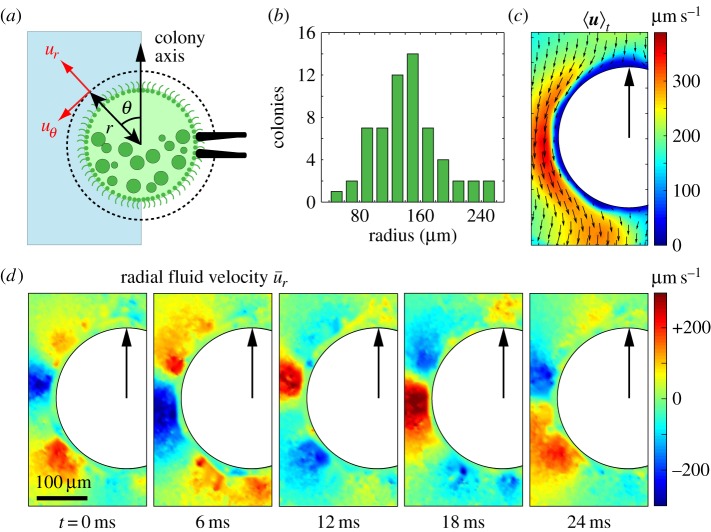
*Volvox* geometry and flows. (*a*) A *Volvox* colony held by a micropipette showing flagellated somatic cells (small dots) and interior daughter colonies (large circles) growing in the posterior half. The dashed line indicates the distance at which the kymographs for the components of the fluid velocity, *u_r_* and *u_*θ*_*, are measured. (*b*) The distribution of radii of *Volvox* colonies used in experiments (total *n* = 60). (*c*) Magnitude (colour) and direction (vector) of the time-averaged flow field obtained using PIV. (*d*) Radial component of the instantaneous fluid velocity, 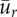, at various times throughout one flagellar beat cycle. The disturbance propagating towards the posterior of the colony is clearly visible.

The projection of the velocity field ***u*** onto the focal plane was visualized by seeding the fluid with 0.5 µm polystyrene microspheres (Invitrogen, USA) at 2 × 10^−4^ volume fraction, and measured using an open source particle image velocimetry (PIV) tool for Matlab (MatPIV) (figure 1). It was then decomposed into radial and tangential components ***u***(*r*, *θ*, *t*) = *u*_*r*_(*r*, *θ*, *t*)***e***_*r*_ + *u*_*θ*_(*r*, *θ*, *t*)***e***_*θ*_, where the local coordinates are defined with respect to the centre of the *Volvox* colony as shown in figure 1*a*. A total of 60 *Volvox* colonies were selected at random from the stock supply, and varied in radius *R* from 48 to 251 µm (mean 144 ± 43 µm), with a distribution shown in figure 1*b*.

**Figure 2.**
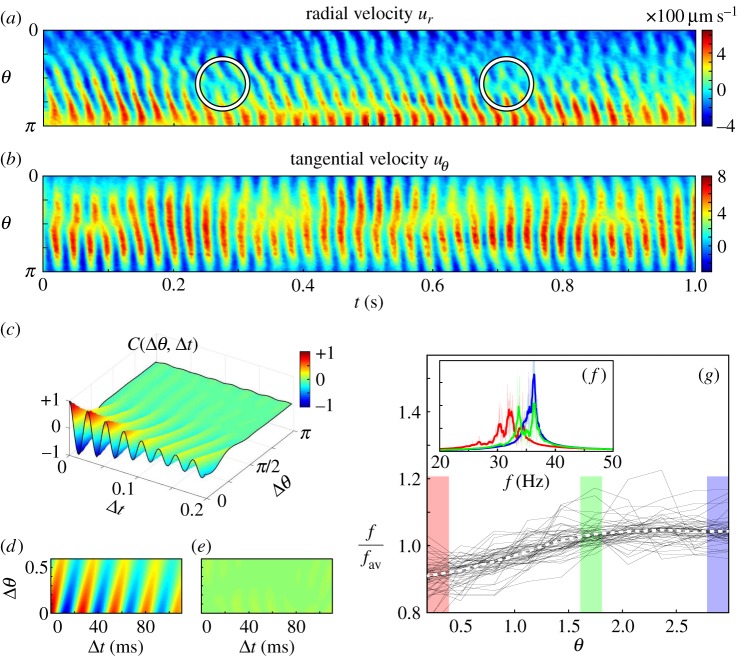
Properties of metachronal waves. (*a*) Radial *u_r_*(r*, *θ*, *t*) and (*b*) tangential *u_*θ*_*(r*, *θ*, *t*) components of the flow field, measured at r* = 1.3 *R*. Phase defects are evidenced by white circles. (*c*) Correlation function *C*(Δ*θ*, Δ*t*) for a single representative *Volvox* colony, (*d*) its fitted correlation function 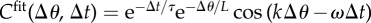 and the (*e*) corresponding error. The scale bar for (*c*–*e*) is the same. (*f*) Power spectrum of the autocorrelation function *C*(0, Δ*t*) (for one *Volvox* colony), calculated for three distinct values of *θ*. (*g*) Average beat frequency as a function of polar angle *θ* for *n* = 60 different colonies (black) as well as the ensemble average (white dotted).

The fluid flow time-averaged over the whole duration *t*_0_ of the movie, 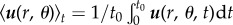 (figure 1*c*), is described accurately by the modes of the ‘squirmer’ expansion of the flow field around a sphere [40–42] modified to take into account the net force exerted by the holding pipette. The results are in agreement with previous measurements on freely swimming colonies [43], and support the hypothesis that flagellar dynamics are the same in held and freely swimming colonies, a fact already established for the closely related unicellular species *Chlamydomonas* [34]. Subtracting the time average from the instantaneous flow field, 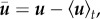 highlights the flow's dependence on the local flagellar phase within the beating cycle. It is this phase that we wish to measure. Figure 2 shows representative kymographs, through the same time interval, for the radial and tangential components of ***u*** at r* = 1.3 *R*. This value of r*, which corresponds to the dashed circle in figure 1*a*, has been found empirically to maximize the kymographs' signals in most experiments.

Temporal variations in the flow are different for the two components (figure 2*a,b*): 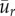 exhibits a wave travelling from the anterior to the posterior pole of the colony (see also figure 1*d*); 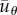 is closer to a standing wave. Such a discrepancy should in fact be expected for a MW propagating along a spherical distribution of flagella as found in *Volvox*: 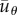 is dominated by the flow induced by the flagella in the equatorial region, which are (approximately) mutually synchronized and are much more numerous than those at other latitudes in the colony. Peaks in 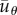 would then happen in the middle of the power stroke of the equatorial flagella, and should correspond to minima in the magnitude of 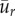 around the equatorial region: this is borne out by the experimental kymographs. We confirmed this hypothesis both by visual inspection of flagellar behaviour and by simulations of arrays of model oscillators (see below). The simulations show clearly that only the component of the velocity perpendicular to the no-slip surface (for us 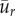) tracks closely the local phase of the oscillators, provided that the phase profile changes sufficiently slowly, as is the case for *Volvox*. This finding is also consistent with the fact that the flow induced by a force normal to a no-slip plane decays faster—and is then more localized—than that from an equivalent tangential force (*r*^−4^ versus *r*^−3^ at constant distance from the surface). 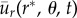 can then be used as a proxy for the local flagellar phase along the beating cycle, eliminating the need to track the motion of individual flagella on the colony's surface. Figure 2*a* corresponds to a MW which propagates in the direction of the flagellar power stroke, called symplectic, and is representative of all the 60 colonies examined (see full experimental dataset for additional results). Direct inspection of several colonies at different orientations confirmed that *Volvox* MWs have only a minimal lateral (diaplectic) component. The average properties of *Volvox* MWs can be quantified [16,29] through the normalized autocorrelation function of 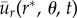 (figure 2*c*), 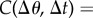 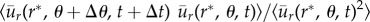 where 〈〉 denotes averaging over all *t* and *θ*. The phenomenological functional form 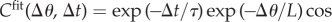 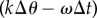 fits the experimental autocorrelation function remarkably well, with average deviation of 0.033 ± 0.011 between the two, across all colonies observed. From these fits we estimate the mean beating frequency *f* = *ω*/2*π* = 1/*T* = 32.3 ± 3.1 Hz; the autocorrelation decay time *τ*/*T* = 3.4 ± 2.0, and decay length *L* = 0.35 ± 0.10; and the mean wavenumber within the population *k* = 4.7 ± 0.9 (twice the number of complete waves along a meridian). *k* is positive in all our experiments, indicating the ubiquitous presence of a symplectic MW which emerges despite the absence, in this species, of any direct intercellular connection between somatic cells.

By averaging MW properties, we necessarily mask brief inhomogeneities in the beating dynamics. These are primarily not random, but rather appear as recurrent defects in the MW pattern, as circled in figure 2*a*. To our knowledge, such defects have never previously been observed experimentally, although a similar behaviour was reported in recent simulations [16]. Reminiscent of so-called frequency plateaus observed in strips of coupled oscillators [44], the defects amount to global slip events, where the phase difference between two groups of oscillators suddenly increases by a full cycle [4,32–34]. In the majority of our experiments, the defects are directly recognizable from the kymograph (figure 2*a*). They consistently appear around the equatorial region, and correspond always to posterior flagella slipping ahead of anterior ones. This results in loss of temporal and spatial correlation of the MW, and it is responsible for the surprisingly short correlation time (*τ*/*T*) and length (*L*) observed. The presence of defects results in the *θ*-dependent average beating frequency seen in figure 2*g*, which could reflect a combination of factors including a physiological bias in the intrinsic flagellar beating frequency across the colony, or the effect of lower drag owing to higher concentration of somatic cells in the colony's posterior. Inspection of the power spectrum of the autocorrelation function of the kymograph signal at *individual* values of *θ* reveals that this transition in average beating frequency has a characteristic structure. Close to the top or to the bottom of the colony, all cells are phase-locked and beat with a single frequency, notably different for the two groups (the main peaks in the red and blue curves in figure 2*f*; the peaks' width is caused by both intrinsic beating variability and measurement error). The transition region, where defects appear, interpolates between these two groups by combining their frequencies. The result is a double peak in the power spectrum (e.g. green double peak in figure 2*f*), where the relative height of the peaks depends on the proximity to one or the other of the phase-locked groups. This phenomenology is characteristic of all the colonies displaying clearly defined phase defects.

Note that the frequency bias would by itself generate a wave propagating in the direction *opposite* to the experimental one, and hence cannot be the fundamental mechanism selecting the direction of the observed MW. In what follows we discuss the behaviour of a minimal model of interacting rotors which spontaneously generates a symplectic MW, and the effect on these dynamics of a large-scale bias as found in figure 2*g*.

## 3. Hydrodynamic model for interacting flagella

**Figure 3.**
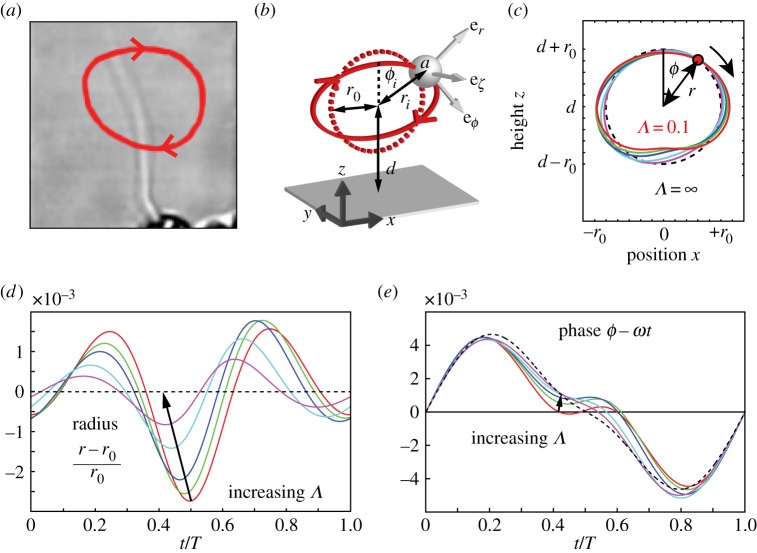
Modelling flagella. (*a*) Tip trajectory over 10 beats of a flagellum of a *Volvox* somatic cell. (*b*) Rotor as a model flagellum: a sphere of radius *a* elastically bound to a circular trajectory of radius *r*_0_ (dashed red) perpendicular to a no-slip plane, driven by a tangential force in the *ϕ*-direction. (*c*) Intrinsic trajectory of an isolated rotor. Steady-state limit cycle of the sphere above the no-slip wall at *z* = 0, for *Λ* = 0.1, 1, 2, 5, 10, ∞. Perturbations from the circular trajectory have been magnified by a factor of 100 so that the shape is clearly visible. The evolution of the (*d*) radius and (*e*) geometric phase through one beating period is also shown.

The surface-mounted somatic cells of *Volvox* are a few tens of micrometres apart, and their flagella are therefore more nearly in the weak-coupling limit than the cilia of historically studied organisms such as *Paramecium*. It is thus appropriate to model the fluid disturbance produced by their operation as a multi-pole expansion [45], of which we will only keep the mode with the slowest spatial decay—the Stokeslet—representing the effect of a point force. This flow is analogous to the far field of a rigid sphere pulled through the fluid. It has recently been shown that representing a *Volvox* flagellum as a single Stokeslet provides an accurate representation of its flow field down to distances of approximately 10 µm, smaller than those typically separating cells within colonies (approx. 20 µm) [4]. High-speed tracking of the flagellar waveform combined with resistive force theory also confirms that the distributed forces associated with the motion can be well represented by a single point force which periodically traverses a closed loop [4]. Inspired by the trajectories of flagellar tips in *Volvox*, and following an approach similar to others [27–29], a beating flagellum will thus be modelled as a small sphere of radius *a* elastically bound to a circular trajectory of radius *r*_0_ by radial and transversal springs of stiffnesses *λ* and *η*, respectively, and driven by a tangential force of magnitude *f*^drive^ (figure 3). The position of the sphere is given by ***x*** = ***x***^0^ + ***s***(*ζ*, *r*, *ϕ*), where ***s*** = (*r* sin(*ϕ*), *ζ*, *r* cos(*ϕ*)). The prescribed trajectory, defined by (*ζ* = 0, *r* = *r*_0_), is perpendicular to a no-slip plane at *z* = 0 representing the surface of *Volvox*, and its centre ***x***^0^ is at a distance *d* from the plane. The proximity of the no-slip boundary causes an asymmetry in the sphere's motion which induces a net flow, thus mimicking power and recovery strokes of real flagella.

The sphere's velocity ***v*** follows the force balance requirement of Stokes flow: 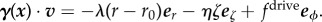 Here 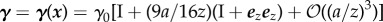 is the friction tensor associated with motion near the no-slip wall [46], and *γ*_0_ = 6*πμa* is the drag on a sphere of radius *a* in an unbounded fluid of viscosity *μ*. Because the drag is not isotropic, the trajectory of an isolated sphere will deviate slightly from the prescribed circle (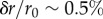 here) to a new limit cycle (*r*(*t*), *ϕ*(*t*), *ζ*(*t*)) of period *T*, which we obtain directly from the simulations for each set of parameters. This limit cycle will represent the unperturbed state of the single rotor. Figure 3 shows the polar coordinates (*r*(*t*), *ϕ*(*t*)) of such limit cycles, for various values of the dimensionless ratio *Λ* = *λd*/*f*^drive^. This parameter governs the deformability of the rotors' orbit: larger (smaller) values of *Λ* mean stiffer (softer) radial recoil compared with the driving force, and result in smaller (larger) deviations from *r* = *r*_0_. For *Volvox* flagella, with bending rigidity *κ* = 4 × 10^−22^ N m^2^ [28] and length *L* ∼ 10 µm, small deflections of the filament would be resisted with an effective spring constant 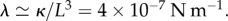 Approximating the driving force as *f*^drive^ = 2*πr*_0_γ_0_/*T*, and taking *a* ∼ 1 µm, *T* ∼ 1/33 s and *d*/*r*_0_ ∼ 1 [29], the normalized spring constant is estimated as *Λ* ∼ 0.1.

From the limit cycle, we define the phase of the rotor, *Φ*(*ϕ*), such that 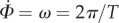 [47]. The presence of the wall implies that the phase *Φ* is different from the angle *ϕ* even for completely rigid orbits. The phase will be useful when studying the dynamics of coupled rotors, since any perturbations from constant evolution of *Φ* will be solely owing to hydrodynamic interactions between different rotors. For a system of *N* rotors, the net hydrodynamic force acting on the *i*^th^ rotor is3.1
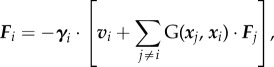
where **G**(***x****_j_*, ***x****_i_*) is the Green's function that couples the different spheres in the presence of the no-slip wall [48,49]. The time evolution of the system of *N* moving spheres is then given by3.2
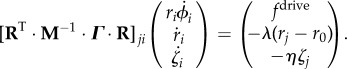
The 3 *N* × 3 *N* matrices ***Γ***, **M** and **R** are defined in terms of their constitutive 3 × 3 blocks 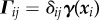, **M**_*ij*_ = *δ*_*ij*_**I** + 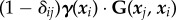 and 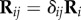, where the columns of the rotation **R***_i_* are given by the vectors 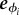, 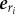 and 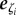, respectively [29].

## 4. Pair synchronization

**Figure 4.**
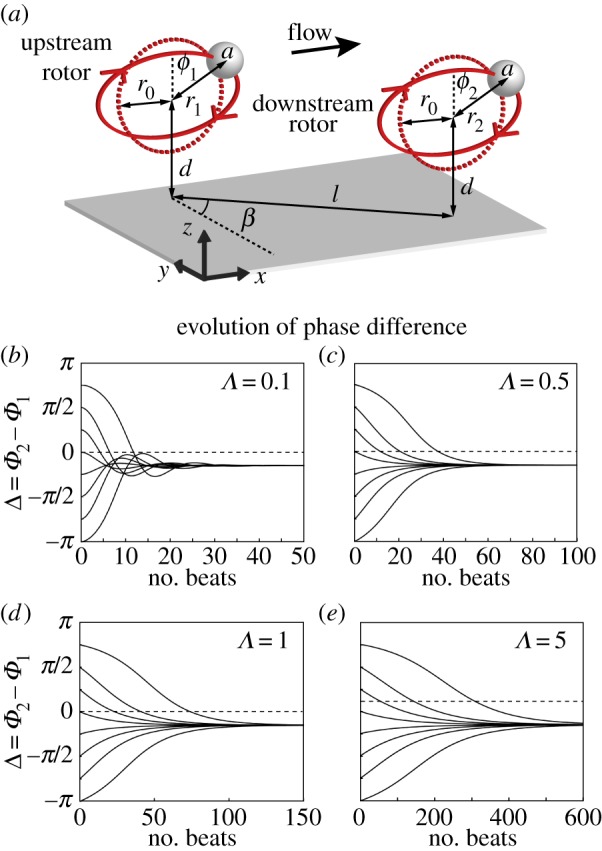
Synchronization dynamics of pairs of rotors. (*a*) Schematic of a pair of interacting rotors, each of which has three spatial degrees of freedom; geometric phase *ϕ_i_*, trajectory radius *r_i_* and out-of-plane displacement *ζ_i_* (not shown). The rotor–rotor spacing *l* and planar angle *β* are depicted. (*b*–*e*) Simulations of interacting pairs of rotors, for various spring stiffness *Λ* = *λd*/*f*^drive^. In each case, the system converges to a steady-state phase difference with Δ < 0, during a time interval that increases with *Λ*. The parameters used are *a*/*d* = 0.01, *r*_0_/*d* = 0.5, *β* = *π*/2, *l*/*d* = 2.

Pair synchronization is the building block of large-scale coordination, and it will be studied here for two identical rotors spaced a distance *l* apart, at an angle *β* in the *x–y* plane (figure 4*a*). For sufficiently stiff springs, hydrodynamic interactions induce only small perturbations around the rotors' limit cycles, and the whole system can then be described just in terms of the two phases (*Φ*_1_, *Φ*_2_). Combining the phases into their sum, *Σ* = *Φ*_2_ + *Φ*_1_, and difference, Δ = *Φ*_2_ − *Φ*_1_, provides a convenient description of the synchronization state of the system, which is simply characterized by Δ(*t*). To develop intuition, we work almost exclusively within this phase-oscillator regime, although simulations use the full dynamical system of equation (3.2).

Figure 4*b–e* shows the evolution of two coupled spheres oriented so that one is directly downstream of the other (*β* = *π*/2), for four different values of *Λ*. In every case, the rotors are able to achieve a phase-locked state by perturbing one another from their limit cycles, and the system converges to a steady state in which Δ = Δ_S_, independently of the initial condition. The fact that Δ_S_ < 0 means that the rotor downstream lags behind the one upstream, a condition which would be naturally expected to produce a symplectic metachronal wave along a strip of rotors. We will see below that this intuition is not necessarily correct.

**Figure 5.**
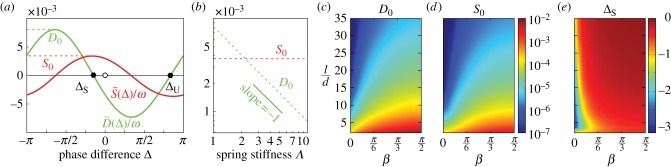
Phase-oscillator reduction. (*a*) Coupling functions 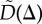 and 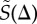 for a pair of interacting spheres. The corresponding parameters are *a*/*d* = 0.01, *r*_0_/*d* = 0.5, *β* = *π*/2, *l*/*d* = 2, *Λ* = *λd*/*f*^drive^ = 1. (*b*) Amplitude of coupling functions for different spring stiffnesses. Computed amplitudes of (*c*) 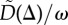 and (*d*) 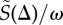, as well as (*e*) the stable equilibrium phase difference Δ_S_, shown as functions of rotor–rotor spacing 2 ≤ *l*/*d* ≤ 35 and offset angle 0 ≤ *β* ≤ *π*/2.

While Δ_S_ depends very little on the precise value of *Λ*, this is not the case for the time scale for phase-locking, which is seen to increase for progressively stiffer rotors. To understand the origin of this dependence, we write the evolution of the phase difference 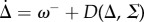 and sum 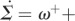 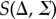 in terms of effective couplings *D* and *S*, where *ω*^±^ = *ω*_2_ ± *ω*_1_. When the phase difference evolves much more slowly than the sum, the equations can be averaged over the characteristic time scale of *Σ* to obtain the time-averaged system:4.1
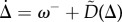
and4.2
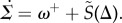
The crossing of curves in figure 4*b* indicates that for that value of *Λ* the evolution of the system is not uniquely determined by Δ: for these parameters the time-averaged equations do not provide a good approximation of the dynamics. Conversely, each panel in figure 4*c*–*e* contains a set of curves that, from a given value of Δ, approach the asymptote in essentially the same manner, supporting the system's description in terms of the single variable Δ. Figure 5*a* shows the coupling functions 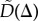 and 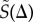 determined numerically from simulations for one set of parameters, and non-dimensionalized by the average beat frequency *ω* (*ω* = *ω*_1_ = *ω*_2_ in this case). These curves are characteristic of the whole parameter range we have studied (0.5 < *Λ* < 10). To great accuracy they are, respectively, −*D*_0_ sin(Δ − Δ_S_) and *S*_0_ cos(Δ − Δ_S_). The system has a stable fixed point at Δ = Δ_S_ < 0, which also corresponds to the maximum in 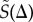: when phase-locked, the rotors increase their average beat frequency owing to a lower effective drag [22,28]. Apart from an increase in speed, this cooperative drag reduction does not influence the synchronization of a single pair, but it will have a major impact on the collective state of larger groups of rotors. Figure 5*b* shows the dependence of the amplitudes *D*_0_ and *S*_0_ on the effective stiffness *Λ*. While *S*_0_ is independent of *Λ*, reflecting the fact that drag reduction is a purely hydrodynamic effect, the amplitude *D*_0_, which governs the convergence towards the stable fixed point, scales as *D*_0_ ∼ *Λ*^−1^ (figure 5*b*). This is reflected in the *Λ*-dependence of the rate of convergence towards phase-locking seen in figure 4*. Stiff* (*D*_0_ < *S*_0_) and *soft* (*D*_0_ > *S*_0_) regimes can be recognized, and the position of the system with respect to this divide can be tuned simply by adjusting the parameter *Λ*, notably by changing the radial stiffness of the orbits. In the asymptotic limit as *Λ* → ∞, phase-locking does not occur (for these parameters)—a phenomenon represented by *D*_0_ → 0. As reported above, we also find that Δ_S_ is independent of *Λ*.

The two most significant parameters governing the spatial configuration of the pair of rotors are the rotor–rotor spacing *l* and the angle *β* (figure 4*a*). Figure 5*c*,*d* shows the dependence of the coupling strengths (*D*_0_, *S*_0_) on (*l*, *β*): both decrease approximately *l*^−3^ with increasing *l*, while at fixed separation they increase monotonically as *β* goes from 0 (side by side) to *π*/2 (aligned downstream). This behaviour follows semi-quantitatively from the (average) magnitude of the flow generated by a single rotor, which in the far field is proportional to sin(*β*)/*l*^3^ [49]. Δ_S_, which is consistently negative, shows instead a much weaker dependence across almost all of the regions of parameter space explored (figure 5*e*). It decays slowly with increasing *l* (approx. *l*^−1^ for *l*/*d* > 10 and *β* ∼ π/2) and is fairly insensitive to changes in *β*, except for a narrow region around *β* = 0 showing strong dependence on the position of the two rotors (Δ_S_ must be an odd function of *β*). The phase lag at synchronization is for the most part almost unaffected by the detailed configuration of the two rotors, in sharp contrast to previous models [25,28]. Simulations for a range of other physical parameters, including sphere size and trajectory radius, show qualitatively identical results.

### 4.1. Influence of a variable driving force

Throughout its beating cycle, it is plausible that a flagellum will exert a changing force on the surrounding fluid and this variation, as opposed to waveform compliance, has been proposed as a major factor leading to phase-locking [27].

**Figure 6.**
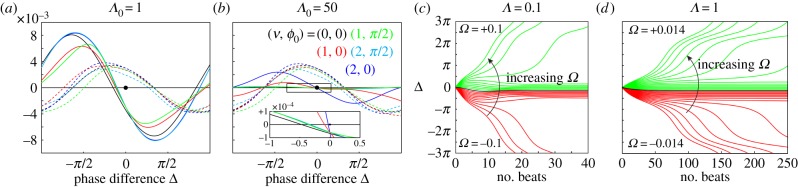
Non-dimensionalized coupling functions 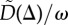 (solid) and 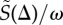 (dotted) for a pair of interacting spheres subject to the driving force 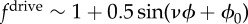 for various values (*ν*, *ϕ*_0_). Plots are shown with (*a*) *Λ*_0_ = 1 and (*b*) *Λ*_0_ = 50. Detuning the rotors: evolution of the phase difference Δ for (*c*) *Λ* = 0.1 and (*d*) *Λ* = 1. Results are shown for various values of *Ω* = 2*ω*^−^/*ω*^+^. Positive and negative values of *Ω* are shown in green and red, respectively, while the black curves represent *Ω* = 0 (identical rotors). All figures correspond to the parameters *a*/*d* = 0.01, *r*_0_/*d* = 0.5, *β* = *π*/2 and *l*/*d* = 2.

Indeed, force modulation can by itself establish synchronization in pairs of model oscillators with rigid trajectories [27], and recent studies [31] have shown that in specific circumstances this can dominate over synchronization mediated by orbit compliance. Here we consider the effect of a modulated driving force *f*^drive^ = *f*_0_(1 + *C* sin(*νϕ* + *ϕ*_0_)) in our system. The average amplitude *f*_0_ is used to define the non-dimensional stiffness *Λ*_0_ = *λd*/*f*_0_. Figure 6 shows the coupling functions 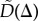 and 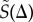 for *C* = 0.5, *ν* ∈ {0, 1, 2} and *ϕ*_0_ ∈ {0, *π*/2}. Note that *v* = 2 corresponds to a class of functional forms highly effective in establishing synchronization in circular rotors [27,31]. For *Λ*_0_ = 1 (and smaller), modulating the driving force has no significant bearing on the results. While changes in *D*_0_ can be significant (≲30%; but *D*_0_ > *S*_0_ always), the difference is mainly far from the phase-locked state, and both Δ_S_ and *S*_0_ do not vary appreciably from their values at constant driving force. In this regime synchronization is dominated by orbit compliance. The corresponding coupling functions for *Λ*_0_ = 50, approximating the rigid case, are presented in figure 6*b. S*_0_ is essentially unchanged, but the phase-locking dynamics are severely altered. Both *D*_0_ and |Δ_S_| have been drastically reduced. The most robust synchronization appears for (*v*, *ϕ*_0_) = (2,0), with a 75% reduction in *D*_0_, and this is accompanied by a lag of just |Δ_S_| ∼ 0.03 (figure 6*b* inset). A system with these characteristics does not generate a MW in a finite chain of rotors, an essential requirement for any realistic model of metachronism. Of course, there may be specific fixed trajectory shapes and force profiles that give rise to an appropriate coupling. However, orbit compliance is capable of facilitating rapid phase-locking in a manner essentially independent of the driving force, in particular for *Λ*_0_ ∼ 0.1. Additional simulations were undertaken with varying rotor geometries, including changes in *β* and *l*, but the results are qualitatively unchanged.

### 4.2. Detuning the beating frequencies

Even within the same group of cells, different flagella will generally have slightly different frequencies. Figure 2*g* shows that this is the case for *Volvox* colonies, with an approximately 10% spread in (apparent) beating frequencies. Here we study the impact of such inhomogeneity on synchronization of a pair of rotors. We focus on frequency differences caused by variations in driving force. The difference between the rotors will be characterized by the fractional frequency difference 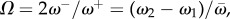 where 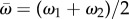 is the mean frequency. Figure 6*c*,*d* shows the evolution of the phase difference Δ for a pair of rotors, beginning at Δ = 0 for *t* = 0, for various values of *Ω*. The curves have been computed for *Λ* = 0.1 and 1, where *Λ* is defined in terms of the average driving force. The parameters corresponding to the smallest *Λ* provide the closest approximation to real *Volvox* cells within this type of model.

Phase-locking is clearly possible for *Ω* in a finite interval around zero, albeit at different values of Δ_S_. Figure 6*c* demonstrates that stable phase-locking can occur for *Λ* = 0.1 even when the rotors possess a |*Ω*| ∼ 6% difference in their intrinsic beat frequencies, which compares well with the measured value of 10% for flagella isolated from *Volvox* [4]. However, for the larger value of *Λ* = 1, the phase-locking is less robust, and the system can support a maximum detuning of only approximately 7% before drifting indefinitely. As 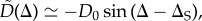 the overall evolution of the phase difference can then be written as 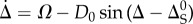 (time is rescaled using the average beating frequency). Stationary points occur for 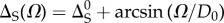, which has a solution only for |*Ω*| ≤ *D*_0_. Because *D*_0_ ∼ *Λ*^−1^, phase-locking will occur below a threshold mismatch |*Ω*|_max_ ∼ *Λ*^−1^. This estimate, based on the time-averaged phase dynamics, is supported by the full hydrodynamic simulations, which show that an order of magnitude increase in *Λ* (from 0.1 to 1) corresponds to an order of magnitude decrease in the threshold detuning (from approx. 6% to approx. 0.7%).

## 5. Group synchronization

### 5.1. Constant driving force

Although necessary, pair synchronization is not a sufficient condition to guarantee that a group of rotors will generate MWs. This fact is evidenced by studying the behaviour of a finite linear chain of rotors, a configuration in which any realistic model of metachronal coordination should be able to produce robust MWs. We describe here the behaviour of linear chains of 30 identical rotors, aligned in the downstream direction, with (*a*/*d*, *r*_0_/*d*, *l*/*d*) = (0.01, 0.5, 2). These are meant to mimic the behaviour of cells along a single meridian of *Volvox*, if they could be observed in isolation. The effective stiffness *Λ* will be varied from 0.1 (*Volvox*-like) to 2. Similar studies were performed for chains of length ranging from 3 to 50 rotors, and 2 ≤ *l*/*d* ≤ 20: the qualitative features of the dynamics remain the same.

**Figure 7.**
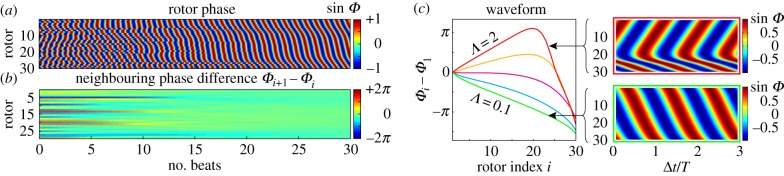
Metachronal wave development. (*a*) The phase along the chain of rotors and (*b*) the neighbouring phase difference *Φ*_*i* +1_ − *Φ*_*i*_ are shown as functions of time. The symplectic MW is established within 15 beats for this value of *Λ* = 0.1. (*c*) Phase profile of an array of 30 spheres after *t*/*T* = 1200 beats. Results are shown for *Λ* = *λd*/*f*^drive^ = 0.1, 0.5, 1, 1.5, 2. All results correspond to parameters *a*/*d* = 0.01, *r*_0_/*d* = 0.5 and *l*/*d* = 2.

Figure 7 shows that for *Λ* = 0.1 a steady symplectic MW develops from random initial conditions within approximately 15 beats. As in a single pair, this phase-locking time scale is stiffness dependent, and within our range of *Λ* it varies from tens to hundreds of beating cycles, independently of initial conditions, eventually diverging for *Λ* → ∞. Figure 7*c* shows the phase profiles along the chains after 1200 beats, representative of the general behaviour. We see that the symplectic MW, defined as *Φ*_*i*+1_ < *Φ*_*i*_ ∀ *i*, morphs into a chevron pattern as the rotors' stiffness is increased. For this configuration, *Λ* ≃ 1 marks the boundary between the two different patterns. Phase-locking into a chevron has been previously reported for similar linear systems [28,50] which were not able, however, to generate also a stable metachronal coordination.

Qualitatively, chevron formation arises from the competition between drag reduction at synchronization and the tendency of neighbouring rotors to phase-lock at a given Δ_S_ ≠ 0. Drag reduction is more pronounced for rotors in the chain's interior, which will tend to lead those at the extremes. This would result in a large fraction of the chain having a phase profile opposite to the one required by metachronal coordination. In the stiff regime (*D*_0_ < *S*_0_), drag reduction dominates, and leads to a chevron-like steady-state phase profile. However, for sufficiently compliant trajectories (*D*_0_ > *S*_0_) the drive towards locking at a given phase lag is strong enough to overcome the effect of drag reduction, and nearest neighbours will synchronize with lags of the same sign as Δ*_S_*. In this system, this results in a symplectic MW. In fact, even within the MW regime, long-range hydrodynamic interactions between rotors at all separations *l* will contribute to determine the steady-state phase lags between nearest neighbours. Because Δ_S_ ∼ *l*^−1^ and D_0_ ∼ *l*^−3^ we can generally expect the magnitude of this phase lag to be smaller than for an isolated pair at the equivalent separation. For example, while a pair with *Λ* = 0.1 would phase-lock with Δ_S_ = −0.47 (other parameters as in figure 7), the average nearest neighbour phase lag that is actually realized in a linear array is significantly less, 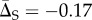. The non-local interactions are clearly important in determining the overall system dynamics. With these parameters, an array of 30 rotors should provide the best approximation to a single meridian of *Volvox* cells from a real colony. Our simulations predict that the system should develop a symplectic MW, indeed observed experimentally, with a wavenumber *k* ≃ 2. Given the simplicity of the model, we believe that this is in good agreement with the experimental average value of *k* = 4.7 ± 0.9.

### 5.2. Inhomogeneous driving forces and defects

**Figure 8.**
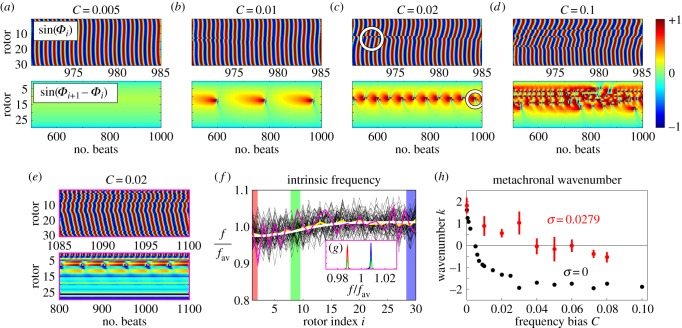
Inhomogeneous driving forces and the appearance of defects. Rotor chains possess a *Volvox*-inspired intrinsic driving force 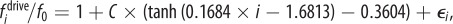, where *ε*_*i*_ = *ε*_*i*_(*σ*) is a random number chosen from a normal distribution with mean of zero and standard deviation *σ*. The frequency bias along the chain is governed by the parameter *C*. (*a*–*d*) Kymographs showing the phase, sin(*Φ*_*i*_), and phase difference, sin(*Φ*_*i*+1_ − *Φ*_*i*_), for various values of *C*, with *σ* = 0 (no polydispersity). An example phase defect is circled in white. (*e*) Kymographs for *C* = 0.02 with *σ* = 0.0279. (*f*) Intrinsic frequency distributions (black) for 60 different realizations with *C* = 0.02 and σ = 0.0279, along with their average (yellow). The magenta curve corresponds to the frequency profile used in (*e*) and the inset (*g*) shows power spectra of sin(*Φ*_*i*_) for oscillators 1 (red), 8–9 (green) and 30 (blue) in this chain. (*h*) Extracted metachronal wavenumber *k*. Simulations without polydispersity (*σ* = 0, black, 19 simulations) and with polydispersity (*σ* = 0.0279, red, 210 simulations) are shown. Other parameters are given by *a*/*d* = 0.01, *r*_0_/*d* = 0.5, *l*/*d* = 2 and *Λ_i_* centred at *Λ* = 0.1.

The experimental results in figure 2*g* demonstrate a spatially dependent intrinsic frequency bias among the flagella in *Volvox*. The average shape of this bias is captured very well by the functional form *f*/*f*_av_ = 1 + *a*[tanh(*b*(*θ* − *c*)) − tanh(*b*(*d* − *c*))], chosen so that the average frequency in *θ* ∈ [0, *π*] is independent of the amplitude *a*. For a chain of 30 rotors, we use the fitted driving force 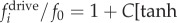 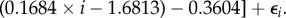 The parameter *f*_0_ is chosen to centre the distribution of resulting effective spring constants 
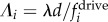
 around *Λ* = 0.1. The amplitude *C* is a parameter controlling the extent of the frequency bias. The value *ε*_*i*_ = *ε*_*i*_(*σ*) is a random number chosen for each rotor from a normal distribution with mean of zero and standard deviation σ, and represents polydispersity in the driving forces along the chain. The appropriate parameters for *Volvox*, *C*_V_ = 0.0724 and *σ*_V_ = 0.0279, were fitted directly from figure 2*g*. The dynamics of rotor chains are explored with 0 ≤ *C* ≤ 0.1 and *σ* ∈ {0, 0.0279}, the results of which are shown in figure 8.

For sufficiently small frequency biases (*C* ≤ 0.007), the system still converges to a phase-locked state, and the only effect is to distort slightly the MW (figure 8*a*). This shows that the MW is robust, a result further confirmed by simulations presented in the electronic supplementary material that explore independently the effects of unbiased polydispersity and a linear frequency bias. For *C* > 0.007, the effect of the bias is the emergence of defects concentrated around the region of maximal slope in the frequency profile (figure 8*b*–*d*). These defects are situated in the central/upper part of the chain—the same region as for the experimental results in figure 2*a*—but exhibit somewhat slower dynamics than the experimental defects, lasting approximately 5–6 beats, compared with 3–4 for the experiments. The defects separate two regions of symplectic metachronal coordination, a small one in the upstream portion of the chain and a larger one in the downstream half. In particular, it is evident that the bottom third of the strip, corresponding to the region of smallest bias, is always coordinated along a symplectic MW. This reflects our experimental observations (figure 2*a*), and it is true for *C* ≤ 0.02. Above that value, this region tends to shrink, going to just six oscillators for *C* = 0.08. For values of *C* large enough to induce defects but ≤0.03 the power spectrum of individual oscillators shows the same qualitative behaviour observed in the experiments: two well-defined frequencies for the top and bottom groups of phase-locked oscillators, and in the middle a transition region characterized by power spectra with a double peak. The relative power carried by the two peaks depends on how close the oscillator is to one or the other of the phase-locked groups. The transition region is where the defects appear, as is the case for the experiments as well. Altogether, these results suggest that *C* = 0.02 is an appropriate choice to mimic experimental behaviour in the model.

The effect of including polydispersity in the driving forces was also investigated, using the measured value from *Volvox* (*σ* = *σ*_*V*_ = 0.0279) in the simulations. For various values of *C*, sets of simulations were conducted, each a different realization of this random polydispersity (210 in total, across nine values of *C*). Figure 8*f* shows the intrinsic frequency profile for 60 different simulations with *C* = 0.02 (black), together with their average (yellow) and the underlying bias (white). The magenta curve corresponds to the kymographs presented in figure 8*e*. Even in the presence of polydispersity, the system displays a symplectic MW on average.

The correlation function used to characterize the mean experimental wave properties is also used here to measure the average metachronal wavenumber in the numerical simulations. For each simulation, the wavenumber *k* was extracted, the results of which are shown in figure 8*h*. Without polydispersity (*σ* = 0), increasing the frequency bias *C* results in a reversal of the metachronal wave, with *k* ∼ −2 for *C* ≥ 0.03. However, including random variations in the intrinsic rotor frequencies (*σ* = 0.0279) increases the wavenumber significantly. For *C* = 0.03, the metachronal wave remains symplectic, with wavenumber *k* ∼ 1. Incorporating polydispersity in this fashion increases the frequency of phase defects, which allow relaxation of the rotor chain to the locally preferred symplectic state. The repeated disruption of this metachronal wave owing to polydispersity in the rotor properties allows the wave to sustain clear symplectic coordination on average, even in the presence of a frequency bias that would otherwise induce a strong reversal of wave direction.

### 5.3. Two-dimensional array of rotors

**Figure 9.**
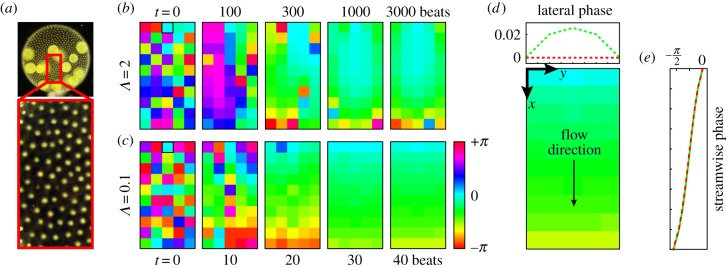
Two-dimensional lattice. (*a*) Photograph of a patch of flagella on the surface of *Volvox* (Goldstein). Phase evolution for a two-dimensional square grid of 50 rotors with (*b*) *Λ* = *λd*/*f*^drive^ = 2 and (*c*) the *Volvox*-like parameter *Λ* = 0.1. In each case, the phase is shown relative to that of the top, central rotor. Other parameters are: orbit radius *r*_0_/*d* = 0.5, rotor–rotor spacing *l*/*d* = 2 and sphere size *a*/*d* = 0.01. Simulations in *b* and *c* have been conducted with free boundary conditions. (*d*) Average lateral and (*e*) streamwise phase profiles for free boundary conditions (green dashed) and periodic boundary conditions in the *x*-direction (red dashed).

Figure 9*a* shows a patch of *Volvox* cells on the colony's surface. Although the distribution is not entirely regular, the intercellular spacing is almost uniform. Following this observation, we will investigate in this section the dynamics of a two-dimensional array of 10 × 5 identical rotors (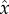 and 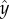 axes, respectively), arranged on a square lattice of constant spacing *l*/*d* = 2. The rotor orbits are parallel to 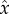, with *r*_0_/*d* = 0.5, *a*/*d* = 0.01 and a radial stiffness 0.1 ≤ *Λ* ≤ 2.

Commencing from random initial phases, the system with *Λ* = 2 moves over several hundred beats towards a phase profile in which the interior rotors lead (figure 9*b*). This is analogous to the profile exhibited for the linear chain in figure 7*c*, whereby drag reduction of interior rotors leads to a chevron phase profile. Figure 9*c* shows that for *Λ* = 0.1 the system develops a symplectic MW within a few tens of cycles with an average phase difference of 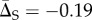 between adjacent rotors with the same *y*-coordinate. This is very similar to the value found for a linear chain (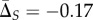), indicating that the one-dimensional configuration is able to capture the salient features of the system. Figure 9*d*,*e* shows the average lateral and streamwise phase profiles, respectively (green dashed curves).

The same behaviour was observed when imposing periodic boundary conditions along 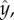 mimicking the periodic arrangement of surface-mounted flagella on *Volvox*. Starting from random initial conditions, all our simulations for *Λ* = 0.1 converged rapidly to a symplectic MW with rotors along the lines of constant *x* locking in-phase. There was almost no measurable effect on the streamwise metachronal wave profile (red dashed curve in figure 9*e*), though the slight phase variations in the *y*-direction were suppressed. This periodic arrangement can also sustain a discrete set of lateral MW components. Our simulations show that an MW with a total phase accumulation of ±2*π* along the periodic direction (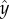) is locally stable, and higher windings ±2*πn* should also be possible, although their domain of stability is likely to shrink with increasing integer *n*. These states did not develop in any of our simulations starting with random initial conditions, so the probability to realize them over the purely symplectic wave is likely to be very small.

## 6. Conclusion

This paper presents a detailed experimental analysis of the global flagellar dynamics on the surface of the multicellular green alga *Volvox carteri*. As a model organism, *Volvox* has the fundamental advantage that its flagella are well separated from each other and hence much more in the low-coupling limit than for any other organism where metachronal coordination has been previously reported (e.g. protozoa, ciliated epithelia). This property allowed us to compare successfully flagellar dynamics in *Volvox* with a minimal model of hydrodynamically coupled elastic rotors. The model develops spontaneously a symplectic MW of a wavenumber that compares well with the one observed experimentally. This MW is robust against inhomogeneities in the rotors' intrinsic frequencies, variations in their spatial arrangement and choice of boundary conditions. These are all essential properties for a plausible model of metachronal coordination. We have shown the existence of MWs which are consistently symplectic on average, but display at the same time characteristic recurrent defects. These might be connected to the observed bias in the distribution of beating frequencies across the colony, which by itself would favour a wave propagating in the opposite direction. We tested this hypothesis within our minimal model, and observed the emergence of recurrent phase defects in the same location and with the same power spectral signature as those observed experimentally. Altogether, these results show that different properties of the underlying oscillators can combine and give rise to much more complex metachronal wave dynamics than previously assumed.

## Supplementary Material

Supplementary materials

## Data accessibility

For data accessibility see www.damtp.cam.ac.uk/user/gold.
