# Supplementary material for: Metachronal waves in the flagellar beating of Volvox and their hydrodynamic origin
Source: J R Soc Interface. 2015 Jul 6;12(108):20141358. doi: 10.1098/rsif.2014.1358 (PMC4528573; doi:10.1098/rsif.2014.1358)
Supplement: Supplementary materials [file rsif20141358supp1.pdf]

Metachronal waves in the flagellar beating of *Volvox* and their hydrodynamic origin: Supplementary materials

Douglas R. Brumley[1,2], Marco Polin[3],
Timothy J. Pedley[1], and Raymond E. Goldstein[1]
[1]Department of Applied Mathematics and Theoretical Physics,
Centre for Mathematical Sciences, University of Cambridge,
Wilberforce Road, Cambridge CB3 0WA, United Kingdom
[2]Department of Civil and Environmental Engineering,
Massachusetts Institute of Technology,
77 Massachusetts Avenue, Cambridge, Massachusetts 02139, USA
[3]Physics Department, University of Warwick,
Gibbet Hill Road, Coventry CV4 7AL, United Kingdom

The results of various simulations are shown here for chains of rotors above a no-slip wall, with inhomogeneities in their driving forces. We explore the effects of introducing a frequency bias of the same functional form as the measured frequency distribution in *Volvox* (see Fig. 2(g)), and also additional polydispersity in the rotor driving forces. This frequency profile in Fig. 2(g) was fitted and for a chain of 30 rotors, the appropriate driving force is given by $f_i^{\text{drive}}/f_0 = 1 + C[\tanh(0.1684 \times i - 1.6813) - 0.3604]$ with $C_V = 0.0724$ and $1 \le i \le 30$ is the rotor index. The parameter f_0 is chosen to centre the distribution of resulting effective spring constants $\Lambda_i = \lambda d/f_i^{\text{drive}}$ around $\Lambda = 0.1$. Figure 2(g) also shows that the frequency profiles for individual *Volvox* colonies are distributed around the mean curve. The standard deviation of this spread was measured to be $\sigma_V = 0.0279$ for *Volvox*. In the following sections, various driving forces and degrees of polydispersity are considered. Multiple realisations of each parameter set are shown, highlighting the general behaviour of each configuration.

Increasing frequency bias $(0.001 \leq C \leq 0.1)$
No polydispersity $(\sigma = 0)$

For small values of C, the shape of the MW is perturbed, yet remains phase-locked and symplectic in nature. For $C > 0.007$ phase defects begin to emerge among the interior rotors. These defects are periodic for $0.007 < C < 0.02$, but for larger values of C, defects emerge at various points in the chain and interact to produce more complex phase dynamics. In every simulation, there exists a region of oscillators at each end which are locally phase-locked.

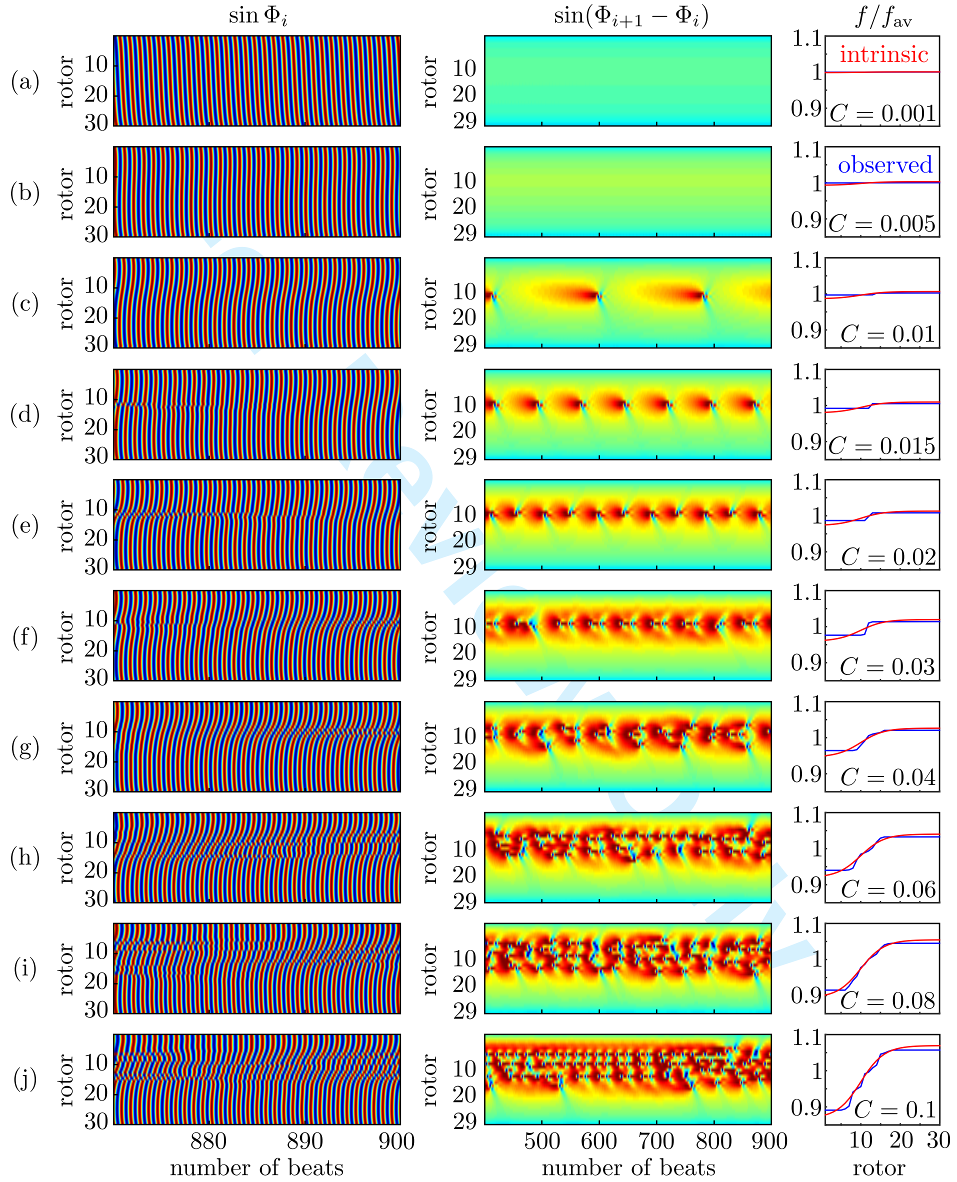

Figure S1: Chains of rotors with an intrinsic frequency bias. The driving force along each chain is given by $f_i^{\rm drive}/f_0 = 1 + C[\tanh(0.1684 \times i - 1.6813) - 0.3604]$ for various values of $C \in [0.001, 0.1]$. The value for *Volvox* is given by $C_V = 0.0724$. Other parameters are given by $\Lambda = 0.1$, $a/d = 0.01$, $r_0/d = 0.5$, $l/d = 2$. The phase profile and phase difference from each simulation are shown, as well as the *intrinsic* rotor frequency profile (red curves) and average *measured* frequency profile from the simulations (blue curves). As the frequency bias is increased, defects in the middle of the chain emerge, becoming more abundant for larger values of C.

Fixed frequency bias ($C = 0.02$)
Polydispersity ($\sigma = 0.0279$)

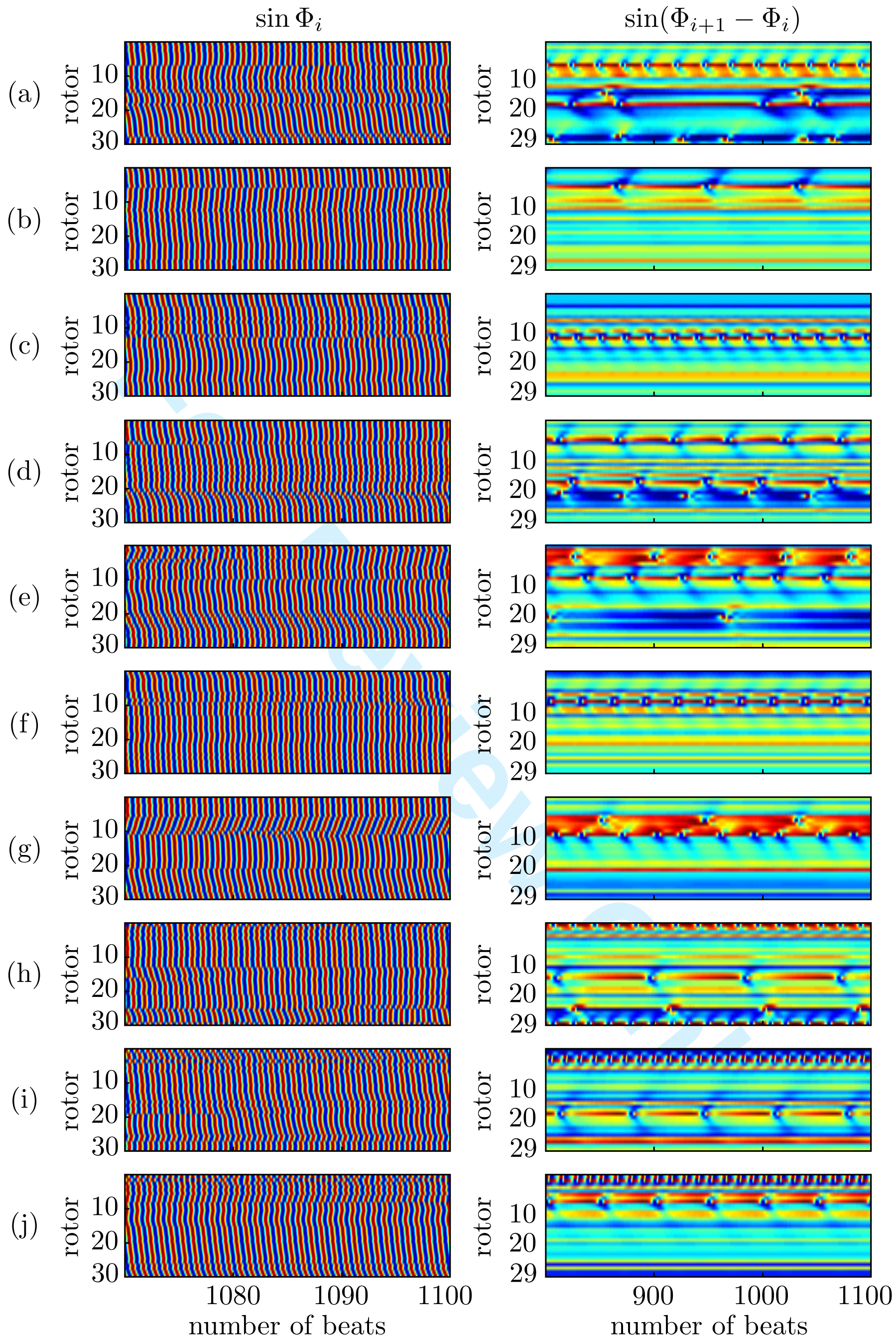

Figure S2: Various realisations of polydispersity. The driving force along each chain is given by $f_i^{\text{drive}}/f_0 = 1 + 0.02[\tanh(0.1684 \times i - 1.6813) - 0.3604] + \epsilon_i$ where $\epsilon_i = \epsilon_i(\sigma)$ is a random number chosen from a normal distribution with mean of zero and standard deviation $\sigma = \sigma_V = 0.0279$. Other parameters are given by $\Lambda = 0.1$, $a/d = 0.01$, $r_0/d = 0.5$, $l/d = 2$. The phase profile and phase difference in each simulation are shown. The colour scale includes -1 (blue) through to $+1$ (red). The results corresponding to 10 of the 60 simulations are shown here, and are representative of the general behaviour observed.

Fixed frequency bias ($C = 0.0724$)
Polydispersity ($\sigma = 0.0279$)

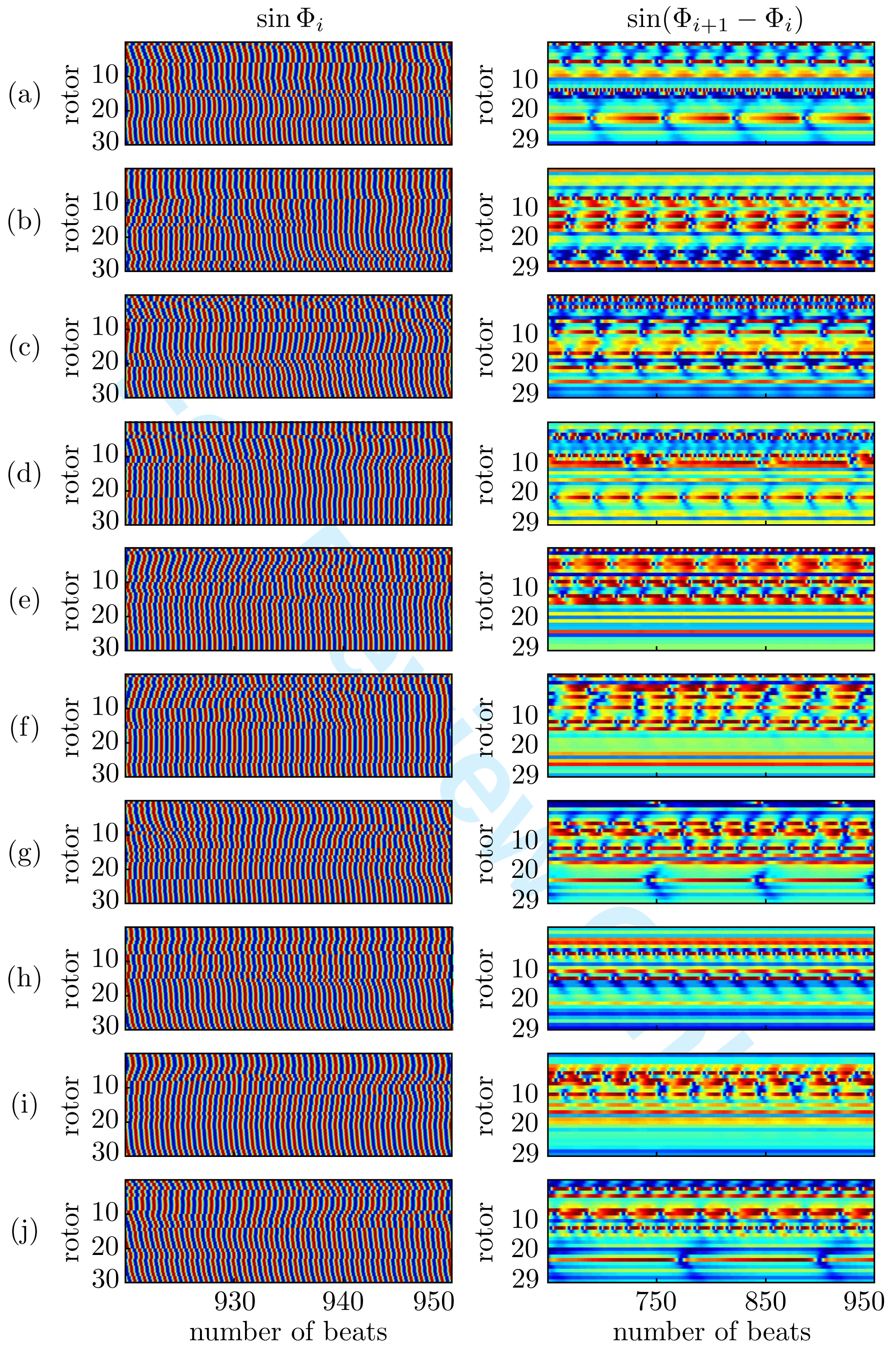

Figure S3: Various realisations of polydispersity. The driving force along each chain is given by $f_i^{\text{drive}}/f_0 = 1 + C[\tanh(0.1684 \times i - 1.6813) - 0.3604] + \epsilon_i$ where $C = C_V = 0.0724$ and $\epsilon_i = \epsilon_i(\sigma)$ is a random number chosen from a normal distribution with mean of zero and standard deviation $\sigma = \sigma_V = 0.0279$. Other parameters are given by $\Lambda = 0.1$, $a/d = 0.01$, $r_0/d = 0.5$, $l/d = 2$. The phase profile and phase difference in each simulation are shown. The colour scale includes -1 (blue) through to $+1$ (red). The results corresponding to 10 of the 60 simulations are shown here, and are representative of the general behaviour observed.

No frequency bias ($C = 0$)
Polydispersity ($\sigma = 0.0279$)

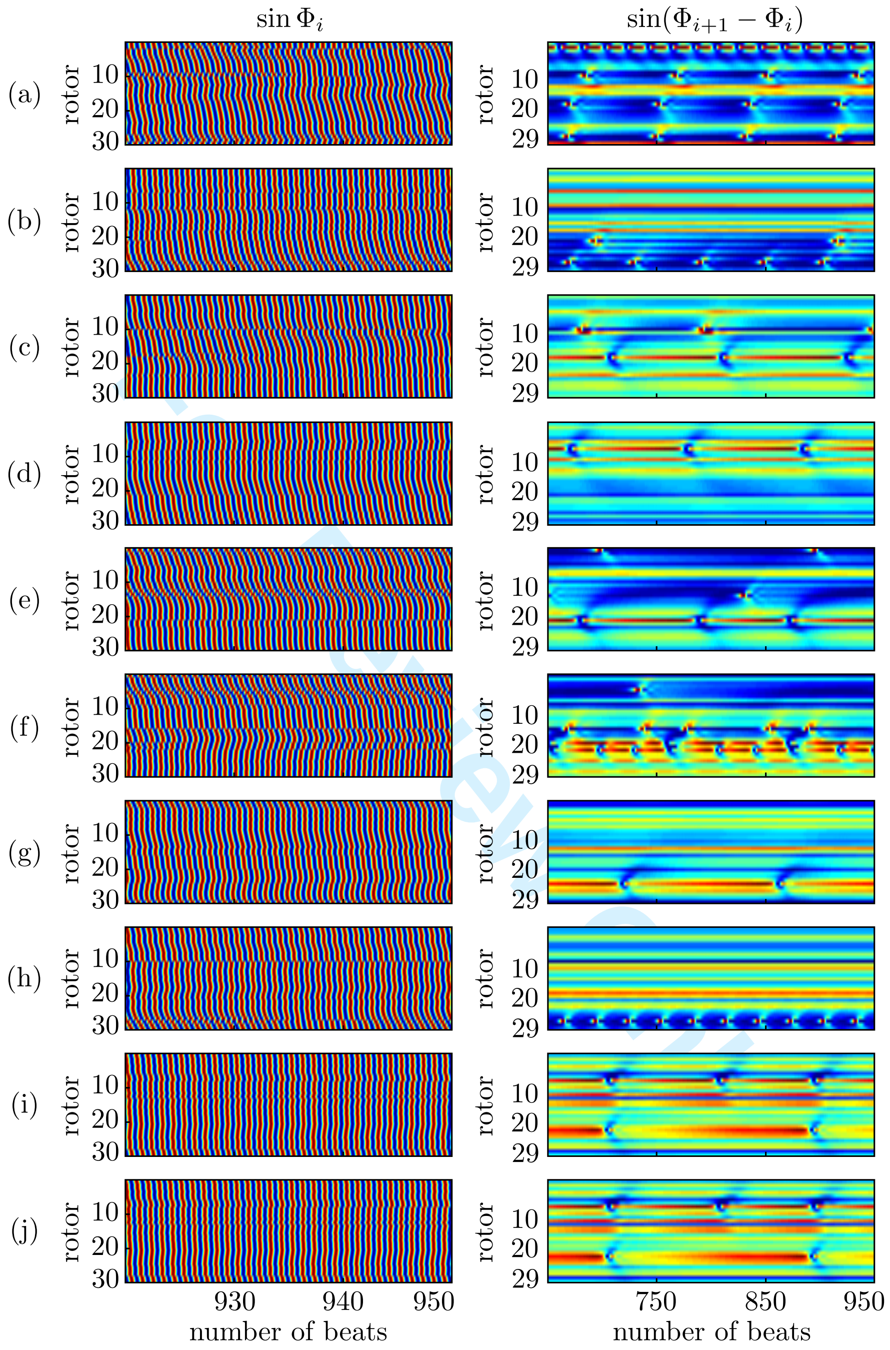

Figure S4: Various realisations of polydispersity. The driving force along each chain is given by $f_i^{\mathrm{drive}}/f_0 = 1 + \epsilon_i$ where $\epsilon_i = \epsilon_i(\sigma)$ is a random number chosen from a normal distribution with mean of zero and standard deviation $\sigma = \sigma_V = 0.0279$. Other parameters are given by $\Lambda = 0.1$, $a/d = 0.01$, $r_0/d = 0.5$, $l/d = 2$. The phase profile and phase difference in each simulation are shown. The colour scale includes -1 (blue) through to $+1$ (red). The results corresponding to 10 of the 30 simulations are shown here, and are representative of the general behaviour observed. Symplectic MWs persist on average, even in the presence of polydispersity.

Correlation parameters

In this section, the correlation parameters are extracted for chains of rotors with varying degrees of polydispersity. The driving force for the ith rotor is taken to be $f_i^{\text{drive}}/f_0 = (1+\epsilon_i)$ where $\epsilon_i = \epsilon_i(\sigma)$, as in Fig. S4, but for various values of σ. The parameter f_0 is chosen to centre the distribution of resulting effective spring constants $\Lambda_i = \lambda d/f_i^{\text{drive}}$ around $\Lambda = 0.1$. With these parameters, the threshold detuning for an isolated pair is $|\Omega|_{\max} \simeq 6\%$. Here we want to investigate the extent to which this applies to MWs, and extract the correlation parameters for the emergent metachronal waves.

Altogether, we performed 150 simulations of arrays of 30 rotors, each run for approximately 3000 cycles starting with random initial conditions. For each simulation, the limit cycle of individual rotors was independently determined, and used to define their phases and beating periods $\{\Phi_i, T_i\}_{i=1}^{30}$. The normalised standard deviation of the set of periods $\psi = \text{std}(\{T_i\}_{i=1}^{30})/T_{\text{av}}$ characterises the variation in the set of beating periods for each simulation. Here T_{av} is the average of $\{T_i\}$. As ψ is increased, the MW that would develop in a system of identical rotors is perturbed initially only slightly ($\psi = 0.009$; rotors globally phase locked), then more heavily ($\psi = 0.028$; rotors locally phase locked), until eventually the MW is realised only on average and any locking is only local ($\psi = 0.052$). Kymographs were used to estimate the average MW properties (T, τ, L, k) for each simulation, as described previously for the experiments.

Figures S5(a-b) show that, generally, both the autocorrelation length and time, L and τ, decrease significantly as ψ is increased. In these plots, the systems with τ larger than the duration of the simulation are coloured green, while the remaining points are shown in blue. The autocorrelation time plot reveals that these two groups cluster around markedly distinct values of τ. Simulations in the first group have $\tau/T \sim 10^6 - 10^7$ and seemingly independent to the value of ψ. They develop a steady MW despite the finite dispersity, but only up to $\psi \sim 3\%$. This value corresponds to the threshold detuning we encountered for two rotors, because for a pair $\psi = \Omega/2$. In the second group, τ decreases with ψ. Figure S5(c) displays the spread of wavenumbers k. The mean wavenumber ($k = 2.1$, dashed line) compares well with the value for identical rotors. With a finite dispersity in the driving forces, the wavenumbers spread in a manner similar to the distribution observed experimentally for *Volvox* (Fig. S5(d)). A few systems at large values of ψ displayed negative wavenumbers ($\sim 6\%$ of the simulations), but overall the simulations show that the present model robustly exhibits symplectic MWs, even with variations in rotor properties.

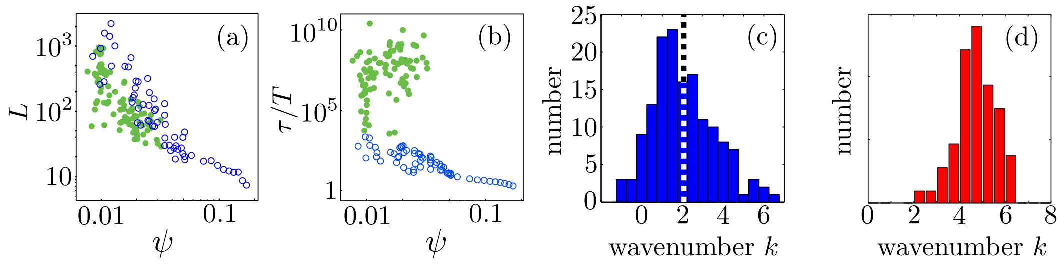

Figure S5: (a) Correlation decay length L and (b) time τ/T scaled by period are shown as functions of the spread in beating periods ψ. (c) Histogram of extracted wavenumbers. Values of ψ are the same as the preceding two plots. Results correspond to 150 simulations of $N = 30$ spheres with $a/d = 0.01$, $r_0/d = 0.5$, $l/d = 2$ and Λ_i centred at $\Lambda = 0.1$. (d) Histogram of wavenumbers in *Volvox* colonies.

Linear frequency bias
No polydispersity ($\sigma = 0$)

To this point, the *Volvox*-inspired intrinsic frequency profile has been used, with the parameter C controlling the extent of this bias along the chain. Chains of rotors with a *linear* bias in their intrinsic frequencies are studied here. The rotors are actuated with a driving force of the form $f_i^{\mathrm{drive}}/f_0 = 1 + D\big[i - (N+1)/2\big]$ for various values of $D \in [-0.0003, +0.0005]$, so that their intrinsic beat frequency varies monotonically along the chain. Figure S6 shows the steady state phase profiles exhibited for a number of values of D. This frequency bias can either enhance ($D < 0$) or reduce ($D > 0$) the slope of the metachronal wave, while still permitting convergence to a steady state. The direction of the metachronal wave is robust, even when the constituent rotors possess a mild opposing frequency bias ($\sim 1\%$ difference between the end rotors).

There are significant qualitative differences between this system, and the one presented in Fig. S1. For the linear profile here, a very small overall frequency difference between the end rotors ($D = +0.0005$) results in suppression of the symplectic metachronal wave. Effective hydrodynamic coupling for each rotor does not extend to more than a few neighbours away, and so the mild linear frequency profile does not provide "weak points" in the coupling. Conversely, the intrinsic frequency profiles in Fig. S1 vary nonlinearly. Defects emerge in the middle of the rotor chain where the neighbouring frequency difference is largest, allowing relaxation to the symplectic metachronal wave.

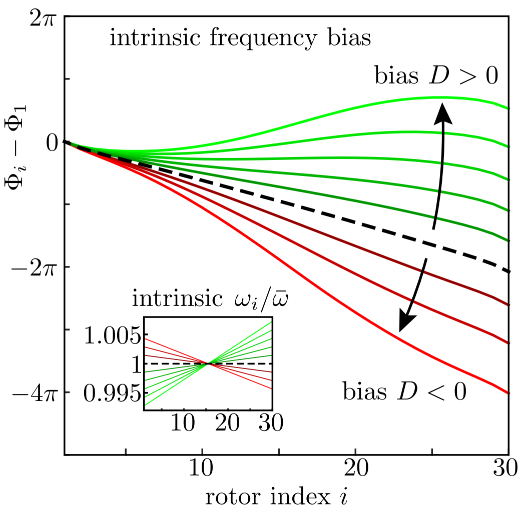

Figure S6: Steady state metachronal wave for chains of rotors with an intrinsic frequency bias. Inset shows the intrinsic frequency of rotors along each chain. The functional form of the driving force is linear, and given by $f_i^{\mathrm{drive}}/f_0 = 1 + D\big[i - (N+1)/2\big]$ for various values of $D \in [-0.0003, +0.0005]$. Other parameters are given by $\Lambda = 0.1$, $a/d = 0.01$, $r_0/d = 0.5$, $l/d = 2$.
